# Human-in-the-loop optimization of wearable device parameters using an EMG-based objective function

**DOI:** 10.1017/wtc.2024.9

**Published:** 2024-11-22

**Authors:** María Alejandra Díaz, Sander De Bock, Philipp Beckerle, Jan Babič, Tom Verstraten, Kevin De Pauw

**Affiliations:** 1 BruBotics, Vrije Universiteit Brussel, Brussels, 1050, Belgium; 2 Human Physiology and Sports Physiotherapy Research Group, Vrije Universiteit Brussel, Brussels, 1050, Belgium; 3 Institute of Autonomous Systems and Mechatronics, Department of Electrical Engineering, Friedrich-Alexander-Universität Erlangen-Nürnberg, Erlangen, 91052, Germany; 4 Department of Artificial Intelligence in Biomedical Engineering, Friedrich-Alexander-Universität Erlangen-Nürnberg, Erlangen, 91052, Germany; 5 Laboratory for Neuromechanics and Biorobotics, Department of Automation, Biocybernetics and Robotics, Jožef Stefan Institute, Ljubljana, 1000, Slovenia; 6 Faculty of Electrical Engineering, University of Ljubljana, Ljubljana, 1000, Slovenia; 7 Robotics and Multibody Mechanics Research Group, Vrije Universiteit Brussel and Flanders Make, Brussels, 1050, Belgium

**Keywords:** human-robot interaction, optimization, performance augmentation, human–in–the–loop optimization

## Abstract

Advancements in wearable robots aim to improve user motion, motor control, and overall experience by minimizing energetic cost (EC). However, EC is challenging to measure and it is typically indirectly estimated through respiratory gas analysis. This study introduces a novel EMG-based objective function that captures individuals’ natural energetic expenditure during walking. The objective function combines information from electromyography (EMG) variables such as intensity and muscle synergies. First, we demonstrate the similarity of the proposed objective function, calculated offline, to the EC during walking. Second, we minimize and validate the EMG-based objective function using an online Bayesian optimization algorithm. The walking step frequency is chosen as the parameter to optimize in both offline and online approaches in order to simplify experiments and facilitate comparisons with related research. Compared to existing studies that use EC as the objective function, results demonstrated that the optimization of the presented objective function reduced the number of iterations and, when compared with gradient descent optimization strategies, also reduced convergence time. Moreover, the algorithm effectively converges toward an optimal step frequency near the user’s preferred frequency, positively influencing EC reduction. The good correlation between the estimated objective function and measured EC highlights its consistency and reliability. Thus, the proposed objective function could potentially optimize lower limb exoskeleton assistance and improve user performance and human–robot interaction without the need for challenging respiratory gas measurements.

## Impact Statement

Wearable devices are important in assisting people, such as patients or older adults, during rehabilitation and everyday activities like walking. Some exoskeletons have been able to reduce the energy cost of walking. However, they require a cumbersome device to quantify it, making it impractical to use in real-life scenarios. Thus, we need to identify a way to assess energetic cost using wearable technologies. To address this, we introduced an EMG-based objective function that captures insights into energetic cost through muscle dynamics and motor coordination. Then, we minimized the proposed objective function online by optimizing walking step frequencies. We found that the EMG-based objective function highly correlates with energetic cost during walking. We also found that our algorithm effectively identifies an optimal step frequency that reduces participants’ energetic cost. These findings will facilitate the customization of the assistance in wearable assistive devices and its application in real situations.

## 1. Introduction

Wearable robots are in continuous development to enhance human movement, motor control, user performance, and overall user experience. The control characteristics of these devices play a key role in defining their behavior and interaction with users. The current focus is on achieving individualized assistance, acknowledging that physiological and neurological differences among individuals affect their response to the same device and assistance (Zhang et al., 2017). Adding to the challenge, as the number of assisted joints increases, so does the complexity of parameter tuning (Zhang et al., 2017; Bryan et al., 2021). As a result, recent advances aim to develop methodologies that enable assistive devices to automatically and continuously customize the assistance (Felt et al., 2015; Zhang et al., 2017). This has resulted in an increasing focus on human-in-the-loop optimization (HILO) strategies, which not only facilitate iterative and automatic tuning of control characteristics (i.e., actuation profile) but also seek to personalize the assistance by minimizing physiological and biomechanical objective functions (Díaz et al., 2022).

Developing a HILO strategy involves defining an objective function that describes user performance and selecting an optimization algorithm capable of minimizing or maximizing the objective function (Díaz et al., 2022). The most widely used objective function for evaluating lower-limb wearable robots during walking is the energetic cost (EC) (Díaz et al., 2022), which displays the overall energy consumption in the body per unit of time (e.g., muscle dynamics, blood circulation, or aerobic processes) (Felt et al., 2015). Measuring EC poses challenges as it relies on indirect calorimetry through an ergospirometric device, resulting in noisy, sparsely sampled, and time-delayed measurements (Felt et al., 2015). The need for a rapid estimation of instantaneous EC is crucial for implementing HILO outside the laboratory. Especially for robotic devices that aim to adjust their assistance based on changes in intensity (i.e., energy requirements) without the need for extended periods of constant activity (Ingraham et al., 2019b). Consequently, researchers have been exploring alternative physiological or biomechanical signals, such as those from portable wearable sensors, to estimate EC more efficiently (Ingraham et al., 2019b; Blake and Wakeling, 2013; Ingraham et al., 2019a). Slade et al. (2019) have shown that muscle activity and vertical ground reaction forces are adequate and rapid energy expenditure estimators during inclined walking. Moreover, Ingraham et al. (2019a) presented multiple physiological and biomechanical signals that highly correlate with EC during various tasks, such as heart rate, ankle acceleration, and the sum of electromyography (EMG) signals. Recently, our group proposed an EMG-based objective function that combines muscle intensity and the variability of muscle synergies to describe the natural energetic behavior of humans walking (Díaz et al., 2022).

Specifically in HILO, EMG has served as an effective objective function due to its instant measurement of muscle effort and higher temporal resolution compared to breath-by-breath assessments (Blake and Wakeling, 2013). Various EMG-based approaches in HILO include 1) decreasing muscle activity from one muscle in response to hip (Xu et al., 2023) or ankle exoskeleton assistance (Wang et al., 2021; Yan et al., 2019), 2) a heuristic approach where muscle activity signals the user’s desire for assistance and guides robot torque (Jackson and Collins, 2019), and 3) a muscle-activity-based cost function tested in an ankle exoskeleton (Han et al., 2021).

EMG research has revealed that these signals represent muscle activity resulting from motoneuronal activation (Farina et al., 2014). However, the musculoskeletal apparatus has many degrees of freedom, making its assessment more complex. Thus, understanding how the central nervous system (CNS) manages the redundancy of the musculoskeletal system is crucial in motor neuroscience and clinical scenarios. The execution of a motor task requires that the CNS combines the information of multiple muscles into an adequate number of motor synergies and coordinates them via a hierarchical neural pathway (Chvatal and Ting, 2013; Hagio and Kouzaki, 2014). These muscle synergies are spatiotemporal building blocks that optimize motor behavior formation (d’Avella et al., 2003). Almost any change to the musculoskeletal system and its coordination patterns leads to an increase in EC (Collins et al., 2015). Consequently, muscle synergies become relevant for understanding changes in EC, providing insights into spatiotemporal modulated activation patterns and analyzing motor task performance (Hagio and Kouzaki, 2014).

Muscle synergies have been used mainly in optimizing upper-limb wearable robots (Garcia-Rosas et al., 2021a, 2021b; Hamaya et al., 2019). In lower limbs, a recent study presented a muscle-synergy-based objective function tested in one participant wearing a hip exoskeleton (Ma et al., 2024). This study shows that muscle synergies and HILO strategies in an exoskeleton can decrease muscle activity and increase motion coordination. However, there is still insufficient research on lower limb wearable robotic devices; the reason might be the complexity of the movement and the number of muscles that need to be modulated for muscle synergies. Latest studies focusing on muscle synergies during walking or balance propose that variations in synergies for different activities directly explain the difference in biomechanical demands of the particular activity (Chvatal and Ting, 2013; Gupta and Agarwal, 2022).

It is widely known that individuals optimize their coordination patterns for factors such as EC, motion performance, and motor control (Zhang et al., 2017). Therefore, we explored EMG signals in our search for physiological measurements that correlate with changes in EC to enhance and speed up HILO. These signals provide valuable insights into EC based on muscle dynamics and coordination patterns derived from muscle synergies (Diaz et al., 2023). Then, the purpose of this study is first to develop an EMG-based objective function that captures individuals’ natural EC when walking at various step frequencies (Diaz et al., 2023). The second objective is to validate and minimize the objective function by optimizing the step frequency using an online Bayesian optimization algorithm. The parameter we optimize is the step frequency, rather than a robotic assistive device because it is easy to prescribe and measure, has a metabolic minimum, and can be easily replicated by other researchers (Felt et al., 2015). For each step frequency, we estimated the EC using the EMG-based objective function and measured it with a standard device. The measured values served as the ground truth for evaluating the accuracy of our objective function. By identifying optimized step frequency values that minimize EC, we expect to demonstrate the efficacy of an EMG-based objective function and lead to its integration into wearable robotic devices, such as lower limb exoskeletons. This means that user performance and human–robot interaction can be optimized without the cumbersome way of quantifying exchanged gasses. It would become feasible to optimize human–robot interaction in real-life scenarios.

## 2. Methodology

**Figure 1.**
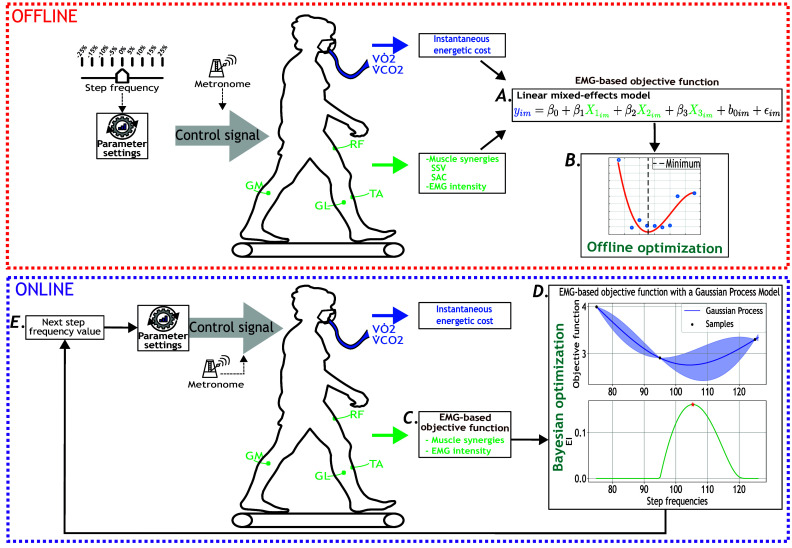
Overview of the offline (red dotted square) and online (purple dotted square) experimental protocols for human-in-the-loop optimization based on the EMG-based cost function. Participants walked at multiple-step frequency values guided by a metronome. Oxygen consumption ( 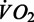 ) and carbon dioxide production ( 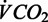 ) were measured using a wearable metabolic system. Surface EMG electrodes recorded bilateral muscle activity from eight muscles: RF, GL, GM, and TA. **A.** EMG-based objective function derived from muscle synergies and muscle intensity, including the similarity of synergy vectors (SSV) and similarity between activation coefficients (SAC). **B.** Offline optimization of the calculated objective function. **C.** Estimation of EC using the EMG-based objective function as a participant walked at a step frequency set by the metronome. **D.** Bayesian optimization updated the step frequency parameter to minimize the estimated cost of walking. EI stands for expected improvement. **E.** New step frequency value given by the Bayesian optimization strategy.

This study consists of two complementary experimental protocols. The first protocol, published in Diaz et al. (2023), focused on identifying the EMG-based objective function that represents individuals’ EC when walking at different step frequencies. In this first stage, we used a steady-state cost mapping to minimize the objective function offline (Felt et al., 2015) (Figure 1A–B). The second protocol is an extended version of the first one where we aimed to minimize the objective function online (Figure 1C–E). Both protocols involved participants walking at prescribed step frequencies (i.e., device parameters) guided by a metronome, which represented the “computer-controlled assistive device” that participants had to follow. Note that for the remainder of this work, the aforementioned first and second protocols are referred to as offline and online, respectively.

This study was conducted following the standards of the Declaration of Helsinki, and the local medical ethical commission (Vrije Universiteit Brussel and University Hospital of Brussel) granted ethical approval.

### 2.1. Experimental setup

For this study, a total of 19 participants were recruited. All participants provided written informed consent before the study. Participants were divided into 2 groups, 10 participants (3 females and 7 males, age = 24.3 ± 2.9 *years*, body mass = 74.3 ± 10.0 *kg*, height = 180.4 ± 9.1 *cm*) completed first the offline optimization experimental protocol. Afterward, the other 9 participants (2 females and 7 males, age = 25.4 ± 3.6 *years*, body mass = 66.8 ± 8.7 *kg*, height = 173.2 ± 5.6 *cm*) completed the online optimization experimental protocol. Both experiments involved participants walking on a treadmill at a constant velocity of 1.25 *m/s* while equipped with a respiratory device and 8 EMG sensors positioned on their lower limbs. At the beginning of each participant’s lab visit, we asked them to walk for 5 minutes at their most comfortable pace to determine their preferred step frequency and serve as a warm-up. Subsequently, the equipment (EMG sensors and respiratory device) was added; participants stood at rest for 5 minutes to evaluate their resting metabolic rate and walked for 5 minutes to assess their metabolic rate when walking at their most comfortable pace.

During each offline protocol, participants completed nine different walking conditions: their preferred step frequency and 25%, 15%, 10%, and 5% below as well as above their preferred step frequency (Felt et al., 2015). Each condition lasted for 5 minutes. We randomized the order of the nine-step frequencies to avoid sequence effects and minimize fatigue.

The online protocol included two walking trials after the standing (resting) and warm-up periods. Participants were given 5 minutes of rest between walking trials. Each trial was a maximum of 24 minutes and consisted of two consecutive phases: initialization and optimization. The initialization phase involved three initial exploration points, which were necessary to initiate the Bayesian optimization algorithm. The optimization phase consisted of one to five Bayesian optimization iterations. Note that the number of iterations depended on how long the proposed algorithm took to converge. For each iteration, participants walked for 3 minutes at the step frequency commanded by the metronome. During the last seconds of each three-minute segment, we used the EMG-based objective function to estimate the EC. Afterward, we used the Bayesian optimization strategy to identify the subject-specific optimal walking cadence. Further details on the optimization strategy are provided in Section 2.4.

Figure 1 shows the setup during data collection for the offline and online protocols. Breath-by-breath rates of oxygen consumption and carbon dioxide production were measured with a respiratory system (Cosmed K5, Rome, Italy). The muscle activity was measured using the Trigno surface EMG system (Delsys Inc., Natick, MA, USA). The muscles selected were based on other research studies in gait optimization (Jackson and Collins, 2019; Bryan et al., 2021). The sensors were located in the following muscles from each leg: rectus femoris (RF), gastrocnemius lateralis (GL), gastrocnemius medialis (GM), and tibialis anterior (TA).

### 2.2. Measured outcomes

In order to propose an objective function that combines motor coordination and muscle effort and adequately explains the altered energy expenditure during walking, we extracted three key measured outcomes: EMG intensity, variability of muscle synergies, and EC. These outcomes form the basis for constructing the proposed EMG-based objective function (later explained in Section 2.3).

#### 2.2.1. Energetic cost

The whole body EC was calculated using the Brockway Equation (1), where *y*(*t*) is respiratory response in Watts, and 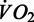 and 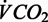 are volumetric flow rates in *mL* * *min*^−1^.(1)

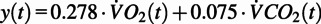



Instantaneous EC was continuously estimated by using a first-order dynamical model described by Selinger and Donelan (2014). This model is commonly used in HILO (Kim et al., 2017; Han et al., 2021; Zhang et al., 2017) to estimate instantaneous EC during non-steady state gait because it considers the delay between measured EC (from respiratory gases) and the body’s instantaneous energy demands.

In line with earlier studies, in the offline optimization protocol, participants were allowed to reach a steady pace during the first minutes of each five-minute walking session (Bryan et al., 2021; Zhang et al., 2017). Afterward, we calculated the instant energy cost using data from the last three minutes.

In the online optimization protocol, we shortened the time per condition since we aim to decrease the time spent in each parameter setting, and therefore the overall time needed for convergence using a HILO methodology. For each three-minute walking condition, participants were allowed to reach a steady state during the initial minute. Once the optimization was completed, the instantaneous EC was calculated over the last two minutes of each walking condition offline. The goal was to compare the output of the EMG-based objective function with the calculated instantaneous EC.

#### 2.2.2. EMG preprocessing

Raw EMG recordings were band-pass filtered (10–450 Hz), full-wave rectified, and low-pass filtered (6 Hz) using a second-order Butterworth filter. For each participant and muscle, the resulting linear envelopes were normalized with respect to the maximum peak amplitude for that muscle. The latest was selected as the maximum value of a 50 ms moving-average window applied to the muscle linear envelopes in each trial (Úbeda et al., 2018). Next, we proceeded to process the EMG signals to calculate both muscle intensity and muscle synergies.

##### EMG intensity

A signal’s power spectral density (PSD) characterizes the distribution of power across frequencies in the signal. The area under the PSD curve represents the power of the signal. Some studies, such as those by Von Tscharner (2000) and Blake and Wakeling (2013), use the term “intensity” to describe the power of the EMG signal. In this context, intensity is defined as a quantitative measure estimating the power of the EMG at time *t* (Von Tscharner, 2000). To obtain the total EMG intensity, one would sum up the EMG intensity across muscles over the time period *t.*

The intensity of the signal was estimated using Welch’s PSD (Hann window length 1 s, overlap 0.60s). The area under the PSD curve was employed to calculate the EMG intensity using Equation 2, where *f* represents the positive frequency and 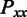 is the intensity per frequency.(2)

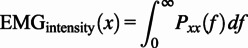



We computed each participant’s overall muscle activity (EMG intensity) for each step frequency at minutes 2 and 3. The total EMG intensity is the combined intensity of all muscles. To find the overall EMG intensity per participant at each step frequency, we averaged the EMG intensity recorded during minutes 2 and 3. Participants were given the initial minute to familiarize themselves with the new step frequency. Subsequently, we focused on processing minutes 2 and 3, intending to minimize the time needed for each set of parameters in future applications.

##### Muscle synergies

Muscle synergies were extracted using a nonnegative matrix factorization (NNMF) from EMG signals. The EMG data were organized in a matrix format, with each row representing the time series of a specific muscle (d’Avella et al., 2003; Chvatal and Ting, 2013; Cheung et al., 2020). NNMF decomposes muscle activities ( 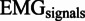 ) into a linear combination of time-invariant synergy vectors ( 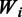 ) and activation coefficients ( 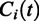 ). Each 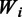 displays the relative contribution of each muscle involved in synergy 𝑖, while the 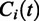 shows changes over time. Four synergies explaining over 90% of signal variance were extracted per condition, aligning with previous findings (Zhao et al., 2021).(3)

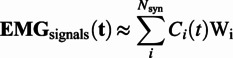



For each step frequency setting, we identified muscle synergies at two specific moments: the start of minutes 2 and 3. In the initial 20 seconds of each minute, we determined the right leg’s stance phases based on the maximum amplitude of the GL. We assumed a gait cycle to initiate with a right stance (indicated by the GL’s maximum amplitude) and conclude after two right stance phases (Diaz et al., 2023). Subsequently, muscle synergies were computed for the average EMG activity of each muscle over the chosen gait cycles. These conditions ensured that participants adapted to the prescribed step frequency for one minute, a complete gait cycle occurred, and the analysis remained consistent across conditions.

Muscle synergies were computed at two distinct time points for each step frequency to measure the variability between these muscle synergies. Lower variability indicates a higher level of coordination. Two metrics were used to demonstrate how synergies change over time: cosine similarity of synergy vectors (SSV) and similarity between activation coefficients (SAC). First, SSV evaluates the similarities between muscle synergy vectors, commonly used for spatial comparison of extracted synergies (Zhao et al., 2021; Gupta and Agarwal, 2022; Nishida et al., 2017). SSV values range from 0 to 1, with the cosine angle extending from 0 to 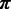 . A smaller angle signifies a higher similarity between two synergy vectors. The mean SSV was calculated across the four synergy vectors at minutes 2 and 3, generating an overall SSV output. Second, we computed the Euclidean distance between activation coefficient curves at each time step to evaluate SAC.

### 2.3. EMG-based objective function

A linear mixed-effects model was employed to combine information from the EMG signals of each participant in the offline experimental protocol. The goal was to create an objective function that closely mirrors the EC variations resulting from changes in step frequency. Linear mixed-effects models extend linear regression for grouped datasets, proving advantageous in studies with repeated measurements, such as ours, involving multiple step frequencies for the same participant.

The proposed model comprises fixed effects (EMG variables) and random effects (participant). Fixed effects, similar to explanatory variables in a regression model, influence the response variable. Random effects are associated with individual experimental units randomly drawn from a population.

To formulate the cost function, it was crucial to calculate three EMG predictor variables (*X*_1_, *X*_2_, *X*_3_). This process involved determining specific aspects of the EMG signals for each participant. SSV was the first predictor variable (*X*_1_), and SAC was the second predictor variable (*X*_2_), both derived from the analysis of muscle synergies. The third predictor variable, EMG intensity (*X*_3_), represented the signal’s power at a particular time t for each muscle. These variables collectively formed the essential inputs for the model to describe the relationship between the response variable (EC) and the independent variables (EMG predictors), accounting for participant-specific coefficients (grouping variable). The model corresponds to Equation 4.(4)

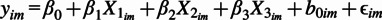

 where 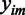 is the observation 𝑖 for each level *m* of grouping variable participant, 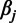 are the fixed-effects coefficients (*j* = 1, 2, 3), 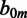 is the random effect for level *m* of the grouping variable participant, and 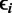 is the observation error for observation 𝑖. The random effects and observation error have a normal distribution.

### 2.4. Human-in-the-loop Bayesian optimization

As mentioned in Section 2.1, human-in-the-loop Bayesian optimization was conducted during the online experimental protocol and was divided into two phases: initialization and optimization. We assessed the estimated EC over three iterations in the initialization phase. The step frequency values from the initialization correspond to the mean, minimum, maximum, and intermediate step frequencies from our preliminary study (Diaz et al., 2023). These initial exploration points are needed to compute an initial posterior distribution before starting the optimization process.

After the initialization, Bayesian optimization was iteratively performed every three minutes using the EMG-based objective function (Figure 1C). First, the posterior distribution of the objective function was estimated as a function of step frequency using a Gaussian process (Figure 1D, top). Then, the next step frequency value to evaluate was the maximum of the expected improvement (Figure 1D, bottom). After, the metronome was updated, and participants were asked to follow the new parameter (Figure 1E). We assumed that Bayesian optimization converged if the new step frequency was in the 1% range from a previously evaluated parameter. Bayesian optimization ended after 24 minutes if the algorithm did not converge.

A Gaussian process was used to model the response surface of the EMG-based objective function. This process was calculated utilizing the mean ( 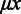 ) and covariance ( 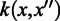 ) (Brochu et al., 2010) with a zero mean. We selected a squared exponential kernel for the covariance function as shown in Equation 5 (Kim et al., 2017).(5)

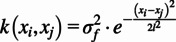

where 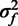 is the variance from the objective function and 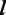 is the length scale parameter (step frequency). The signal variance captures the overall magnitude of the cost function variation, and the length scales capture the sensitivity of the objective function with respect to changes in step frequency. Considering that the estimated EC 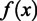 has additive, independent, and identically distributed noise, the samples can be expressed as(6)

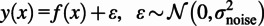

where 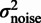 is the noise variance and a hyperparameter guiding the behavior of the model. Given the Gaussian process prior and data set, **D**, the posterior estimated EC distribution was calculated for a step frequency parameter, x_*_, as 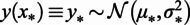 . The mean and variance are calculated as explained by Kim et al. (2017).

To acquire the next step frequency parameter, we used the expected improvement (EI), also known as the acquisition function, which balanced the exploration and exploitation of the Bayesian optimization algorithm. EI selected the next parameter by calculating the expected reduction in the objective function over the step frequencies previously evaluated using Gaussian process posterior distribution (Kim et al., 2017; Ding et al., 2018). The next parameter was then calculated using Equation 7.(7)

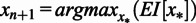



The *argmax* function identifies the step frequency that corresponds to the maximum EI value within the parameter range ( 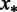 ). The newly selected parameter is then sent to the metronome.

### 2.5. Data analysis

#### 2.5.1. Offline optimization

Following data collection from the offline protocol, we could continue to the offline optimization phase. Here, we constructed a linear mixed-effects model to estimate ECs for each participant at step frequencies that range between −25% and 25% of their preferred step frequency. Employing the Steady-State Cost Mapping method, we fitted a cubic polynomial to steady-state measures across the different step frequency settings, identifying the step frequency that minimized EC. We calculated the sum of squared errors (SSE) and 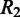 for each participant’s fitted cubic polynomial on estimated EC across step frequencies. Finally, we conducted a linear regression to compare measured and estimated EC values. These steps enhanced the robustness of our model and provided insights into its predictive accuracy across multiple participants and step frequencies.

#### 2.5.2. Online optimization

After completing the online optimization, we compared the estimated EC, derived from the EMG-based objective function with the measured EC obtained using the ergospirometric device. This analysis exclusively utilized data from the online optimization protocol. Both estimated and measured ECs underwent normalization using the min-max method, transforming the data range from 0 to 1 for ease of comparison.

We used a mixed-effects model to assess the relation between measured ( 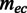 ) and estimated ( 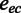 ) EC. This approach accounts for the nonindependence of observations within the same participant by incorporating each participant as a random effect: 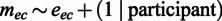 . Due to a very low variance component for the random effects ( 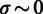 ), which indicates minimal individual differences, we conducted a linear regression analysis. This additional analysis allowed us to assess the correlation between measured and estimated ECs by quantifying how much of the variance in measured EC is explained by estimated EC.

Additionally, we computed the error between the preferred and optimal step frequency values to evaluate the effectiveness and accuracy of the proposed objective function. Furthermore, we compared the measured EC at the optimal step frequency determined by the Bayesian Optimization with the EC of participants walking at their preferred step frequency.

## 3. Results

### 3.1. EMG-based objective function

**Figure 2.**
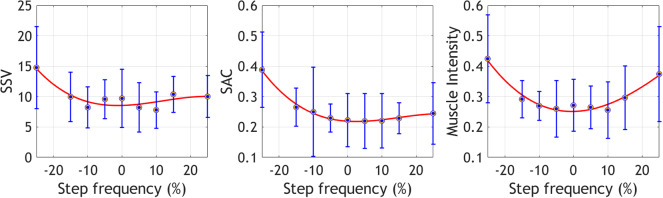
Mean ± standard deviation of the selected EMG variables at the different step frequencies. These metrics were collected during the offline optimization protocol.

Figure 2 displays the mean values for the EMG variables across step frequencies. EMG outputs for the preferred and near step frequencies (−5% to 5%) exhibit the lowest values in most variables, except for SSV, which also has low values at −10% and 10% of the preferred frequency. The highest and lowest step frequencies show the highest outputs across variables, suggesting a similar behavior to EC in the selected EMG variables.

**Figure 3.**
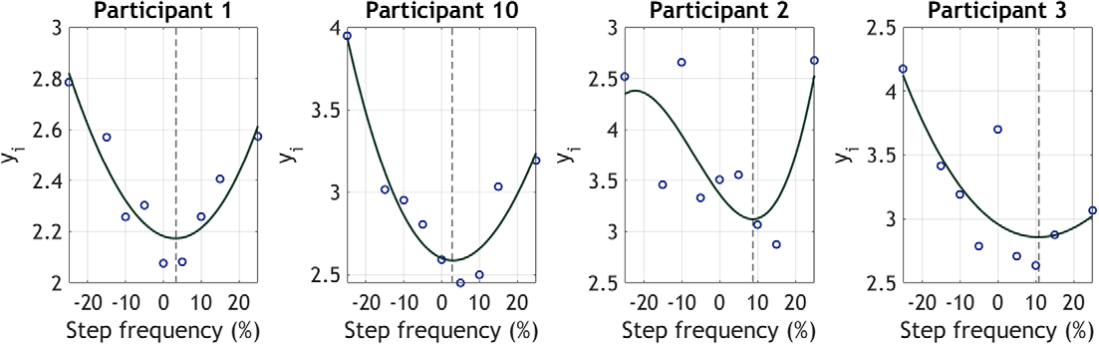
Illustration of the model fitting process for four participants (the best and worst model fittings are included). Each panel shows EC estimates at step frequencies ranging from −25% to 25% of each subject’s preferred step frequency (see Equation 4). A third-order polynomial was used (steady-state cost mapping algorithm) to determine the percentage step frequency that yields the minimum 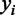 . The minimum value from the curve is indicated with dotted lines.

Table 1 shows that the fitted cubic polynomial over the estimated EC (*yi*) was good for most participants with 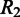 values above 0.7 and low SSE values (*SSE* < 0.3). Figure 3 presents the estimated EC ( 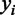 ) for four participants at each step frequency, calculated using Equation 4. The figure also includes the fitted cubic polynomial model derived from the steady-state cost mapping algorithm. The participants selected are those with the best and worst model fits, based on Table 1. The curves illustrate that the estimated ( 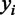 ) increases as the step frequency deviates from the preferred.

**Table 1.** Sum squared error (SSE) and 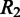 for each participant’s fitted cubic polynomial on estimated EC across step frequencies. Results derived from the offline optimization protocol

Participant	SSE	R2
1	0.06	0.87
2	0.14	0.92
3	0.25	0.59
4	0.73	0.65
5	0.25	0.91
6	0.11	0.94
7	0.10	0.76
8	0.10	0.95
9	0.31	0.70
10	0.06	0.76

The linear-mixed effects model has 90 observations, 4 fixed-effects coefficients, 10 random effects coefficients, and 2 covariance parameters. Analysis of Table 2 revealed significant effects of the intercept, X_2_, and X_3_ (*p* − *values* ≤ 0.05) on EC. X_1_ was retained in the model to improve prediction accuracy. The model’s mean squared error was 0.12.

**Table 2.** Fixed-effects coefficients (95% confidence intervals)

Name	Estimate	SE	tStat	p value	Lower	Upper
**Intercept**	1.193	0.243	4.909	<0.001	0.710	1.676
**X1**	0.008	0.013	0.6587	0.5118	−0.017	0.0342
**X2**	2.015	0.613	3.287	0.001	0.796	3.233
**X3**	3.396	0.501	6.769	<0.001	2.399	4.394

**Figure 4.**
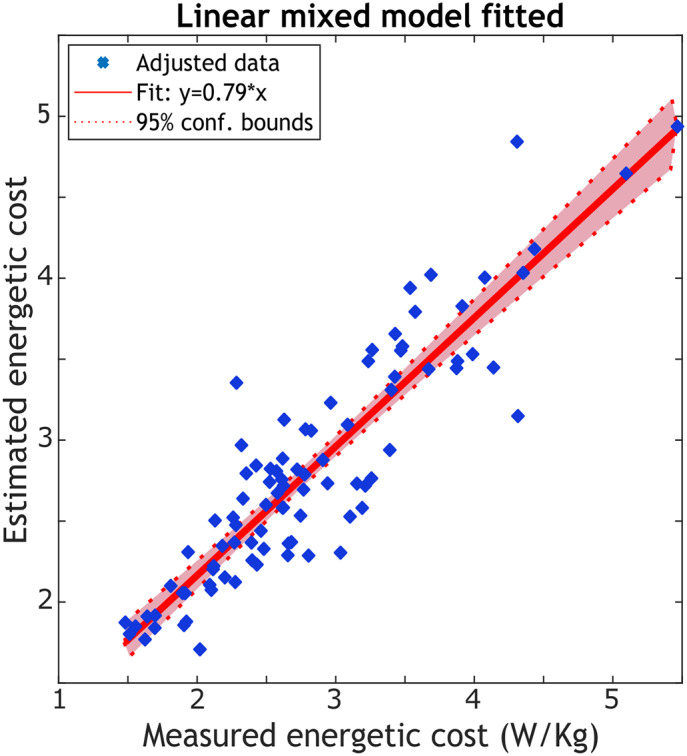
Estimation of the energetic cost after fitting the model to the data set from the offline optimization protocol using Equation 4. The R-squared from the linear regression is 0.82.

The fixed-effects coefficients 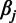 highlighted in blue in Table 2 are the relevant values used to estimate 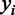 using descriptive EMG variables as inputs. In Figure 4, the relationship between the observed response (measured EC) and the fitted response (estimated EC) is illustrated, with an 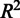 of 0.82 indicating a well-fitting model.

**Figure 5.**
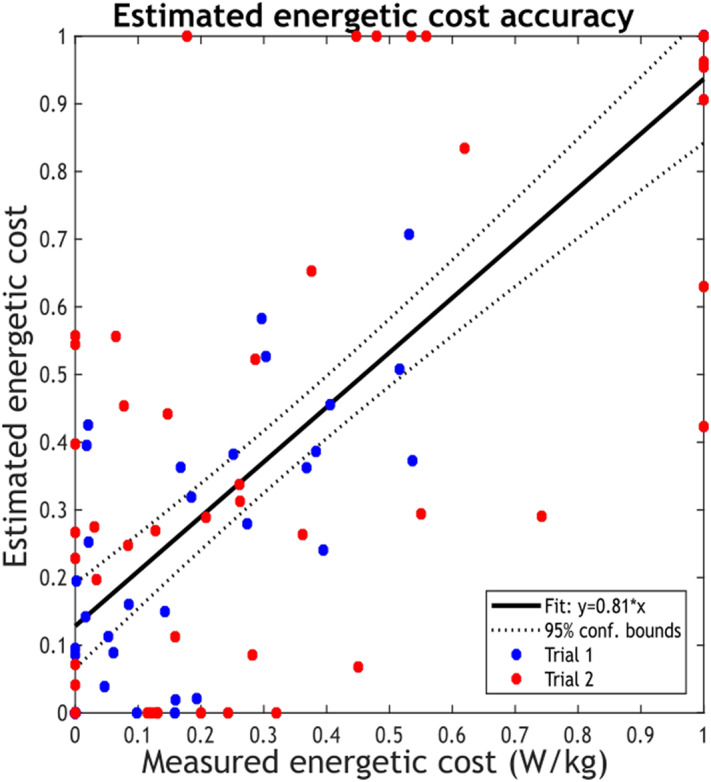
Correlation between normalized energetic cost (Measured) and normalized EMG-based cost function (Estimated). The R-squared from the linear regression is 0.64.

### 3.2. Online Bayesian optimization

Each participant completed the online protocol twice. Here, we refer to each iteration as trial 1 and trial 2, respectively. The mixed-effects model analysis, used to compare measured and estimated ECs across participants, showed a significant fixed effect of estimated EC on measured EC (*p*_values_ < 0.001), with an effect size estimate of 0.79. This confirms a good positive relationship between the estimated and measured ECs across participants, adjusted for individual variations. In the linear regression analysis, the 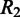 was 0.64, confirming the good correlation between the measured and estimated EC.

**Figure 6.**
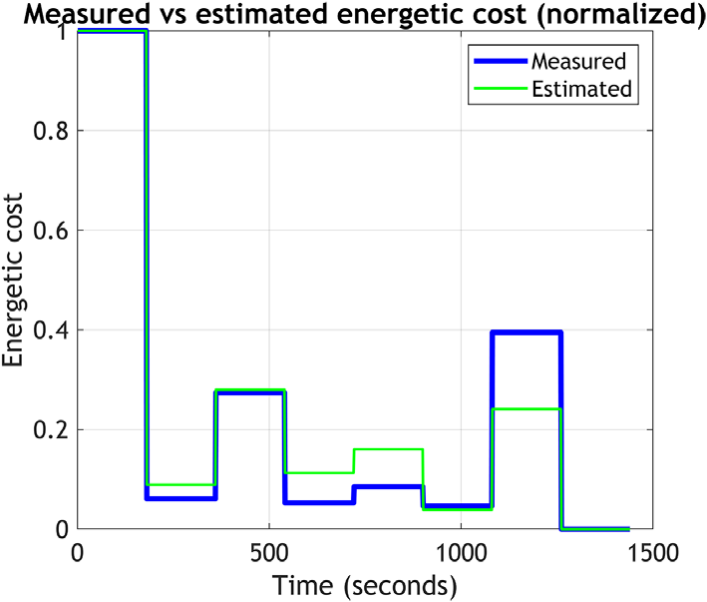
Comparison of measured and estimated energetic cost (EC) during walking at different step frequencies for one representative participant. Each step frequency was maintained for 180 seconds. The Bayesian optimization was initialized with the first three values, and subsequent parameters were determined by the optimization algorithm until its convergence to a minimum.

Figure 6 illustrates an example of the general behavior of the objective function throughout an experiment and how it relates to the measured EC for one representative participant. For this example, the correlation between the output from the EMG-based objective function and the estimated EC was highly significant (Pearson correlation = 0.97).

Moreover, to assess the consistency of the EMG-based cost function, each participant underwent the experiment twice with identical initialization parameters for both trials. Then, the mean squared error between the initialization outputs of both trials was calculated, resulting in an error of 0.198.

Table 3 provides information on the convergence time for each trial and the percentage error between the preferred and optimal step frequencies indicated by the objective function. The average time to converge per trial was 15 and 16 minutes, respectively. Similarly, the mean number of iterations was 5. The percentage errors fall within −6% to 6%, and the mean error was 4%.

**Table 3.** Time to converge and % error between the preferred step frequency (PSF) and the optimal parameter given by the optimization are presented for the first and second trials

Participant	1	2	3	4	5	6	7	8	9
**% Error PSF**	−2.88	4.12	−1.79	6.00	5.00	2.73	4.63	0.94	4.55
	−3.85	−6.19	1.79	−3.00	−4.00	6.36	−5.56	−3.77	−1.82
**Time to converge (min)**	15	15	12	12	12	12	24	12	21
	15	15	12	18	15	21	15	12	24

The optimization method resulted in an approximate 12% reduction in EC for five participants in both the first and second trials, compared to their EC at their preferred step frequency. This indicates that participants experienced lower EC when walking at the optimized step frequency than at their preferred step frequency.

## 4. Discussion

In this study, we developed and validated an EMG-based objective function designed to capture the natural EC of walking at various step frequencies. Our analysis demonstrates that variations in EMG signal intensity and muscle synergies are effectively correlated with changes in EC across different walking cadences. Using this information, the proposed model determines the optimal step frequency for individuals, enhancing both performance and energy efficiency. Initially evaluated in an offline setting, the model was subsequently implemented in an online framework where it was validated through an online Bayesian optimization strategy. The optimization successfully determined optimal step frequencies for participants with notable consistency and accuracy.

In the offline optimization, we used a Steady-State Cost Mapping, an established method that fits a cubic polynomial to steady-state measures at various parameter settings. This method, previously utilized by Felt et al. (2015) to assess ECs across multiple step frequencies, effectively characterizes the EMG-based objective function at different step frequencies across participants in our study. However, an offline model is time-consuming for participants and does not allow for an automated tuning process. Thus, we validated our preliminary results with an online Bayesian optimization strategy commonly used in HILO studies because it is well suited for optimizing noisy and expensive-to-evaluate objective functions such as energetic cost. While a direct comparison between the offline and online estimations of the objective function is challenging due to variations in protocol characteristics (such as the number of iterations, time per iteration, and step frequencies), it is important to highlight the robustness of our model. In the offline approach, the linear mixed model explained 82% of the dataset’s variance in measured metabolic cost. Despite the limitations imposed by the small offline data set (*n* = 10), we anticipated that the model could not be generalized well; thus, we expected a lower performance in the online optimization. Nevertheless, even with an R-squared of 0.65 in the online optimization, our model demonstrates a good correlation and performance, underlying its reliability and effectiveness in capturing individuals’ natural energetic optimization strategy while walking at different step frequencies.

Muscle activity has proven pivotal in predicting the metabolic cost of walking (Ingraham et al., 2019a; Blake and Wakeling, 2013; Ingraham et al., 2017) and has shown relevant results as a performance metric in HILO (Zhang et al., 2017; Jackson and Collins, 2019; Han et al., 2021). While previous studies focused on muscle activity intensity alone, our preliminary results highlighted the potential of including muscle synergies variability in an objective function (Diaz et al., 2023). The optimal step frequency minimizes muscle synergies variability, restoring participants’ motor coordination, and reduces overall muscle activity intensity. This leads to a more energy-efficient walking pattern, contributing to overall energy cost reduction. Our results align with Felt et al. (2015) and Kim et al. (2017), who were able to estimate optimal step frequency and minimize instantaneous metabolic cost with different optimization strategies. However, our results demonstrated a positive trend in identifying a step frequency value around 5% of their preferred that output a lower energetic cost than the one calculated when walking at their preferred step frequency.

It is hard to explain what guides users’ preferences; it can be related to balance, comfort, or energy requirements (Ingraham et al., 2022). The association between kinematic synergy and user preference suggests that participants exhibit higher coordination and, thus, reduced EC when guided by a metronome near their preferred step frequency. Including user preference in the design of such objective functions will be interesting since it is known as a meta-criterion that considers factors that are challenging to quantify but significant to the user experience (Ingraham et al., 2023). Some studies have focused on understanding the impact of wearable device assistance on motor control and coordination. Steele et al. (2017) studied the effects of an ankle exoskeleton assistance in coordination patterns when healthy participants walk. The authors showed that the complexity of the muscle coordination patterns (i.e., synergy vectors) was similar during unassisted and assisted walking, but wearing the exoskeleton impacted the activation coefficients (i.e., temporal activation patterns). The results presented in this study are comparable with the ones from Steele because we highlight changes in activation coefficient variability (referred to as SAC) with varying step frequencies (i.e., device parameters) during walking. Other researchers have also established connections between muscle activation patterns and metabolic cost when the assistance from an ankle exoskeleton varies (Jeong et al., 2023). These results collectively underscore the potential applicability of the presented objective function in enhancing lower limb exoskeletons.

In HILO, only one study has used muscle synergies to optimize the assistance from lower limb wearable devices. In that study, Ma et al. (2024) optimized the assistance of a hip exoskeleton based on a synergy similarity coefficient that compares the muscle synergies from a participant walking with a set of control parameters with a reference synergy calculated when participants walked without the exoskeleton. The challenge of muscle synergies is how to quantify changes properly, so researchers have used a reference to facilitate comparison and present a metric that can assess performance. However, calculating the correct reference adds to the challenge, as it relies on the dataset used for calculation. Our study avoids the need for a reference, which is hard to have available in some patient populations. Additionally, the muscle synergies variability could be an interesting metric to assess performance in other scenarios.

This study employed a limited population of young and healthy participants. However, we aim to expand the data set to enhance algorithm robustness, decrease converging time, and extend the application to various motion tasks.

## 5. Conclusion

This study highlights the relation between an EMG-based objective function, that relies on muscle intensity and muscle synergies coordination, and the EC of humans walking at different step frequencies. The consistent performance of the EMG-based objective function output across participants indicates the potential of using this proposal to achieve a practically feasible HILO of powered wearable devices. Consequently, we expect to extend this application further to lower limb exoskeletons and explore how effectively lower-limb wearable devices can be optimized using the proposed objective function.

## Data Availability

The data that support the findings of this study are available on request from the corresponding author M.A.D.P.
